# Adoptive Cellular Immunotherapy in Metastatic Renal Cell Carcinoma: A Systematic Review and Meta-Analysis

**DOI:** 10.1371/journal.pone.0062847

**Published:** 2013-05-07

**Authors:** Xiaoyi Tang, Ting Liu, Xuefeng Zang, Hao Liu, Danhong Wang, Hu Chen, Bin Zhang

**Affiliations:** 1 Department of Hematopoietic Stem Cell Transplantation, Affiliated Hospital of Academy of Military Medical Sciences, Beijing, China; 2 Cell and Gene Therapy Center, Academy of Military Medical Sciences, Beijing, China; 3 Military Postgraduate Medical College, General Hospital of Chinese People’s Liberation Army, Beijing, China; Shanghai Jiao Tong University School of Medicine, China

## Abstract

**Purpose:**

Metastatic renal cell carcinoma (mRCC), as one of the most immunogenic tumors has been the focus of adoptive cellular immunotherapy (ACI), but the effects of ACI on objective response and survival in patients with mRCC are still controversial. Therefore, a systematic review and meta-analysis was performed to address this issue.

**Methods:**

A search was conducted in the PubMed database for randomized clinical trials (RCTs) with ACI in mRCC. All included articles in this study were assessed according to the selection criteria and were divided into two groups: ACI *versus* no ACI. Outcomes were toxicity, objective response, 1-, 3- and 5-year survival. Risk ratio (RR) and 95% confidence intervals (CI) were calculated using a fixed-effects meta-analysis. Heterogeneity was measured by value of I^2^ or P.

**Results:**

4 studies (469 patients) were included. Most of ACI-related adverse reactions were grade 1 or 2 and reversible. ACI provided significant benefit in terms of objective response (RR = 1.65; 95% CI, 1.15 to 2.38; P = 0.007, I^2^ = 49%), 1-year survival (RR = 1.30; 95% CI, 1.12 to 1.52; P = 0.0008, I^2^ = 0%), 3-year survival (RR = 2.76; 95% CI, 1.85 to 4.14; P<0.00001, I^2^ = 46%) and 5-year survival (RR = 2.42; 95% CI, 1.21 to 4.83; P = 0.01, I^2^ = 28%).

**Conclusions:**

ACI may be a safe and effective treatment for improving objective response, 1-, 3- and 5-year survival in patients with mRCC. Besides, five obstacles for ACI, including high degree of personalization, unsuitable WHO/RECIST response criteria, inadequate identification of tumor-associated antigens (TAAs), lack of effective combination treatments and less attention paid to the quality of ACI products, should be overcome during the successful development of more potent ACI for cancer in the future.

## Introduction

Cancer immunotherapy attempts to harness the power and specificity of the immune system to fight against cancer and has made two major breakthroughs (Sipulecel-T and Ipilimumab) [1]–[3]. Adoptive cellular immunotherapy (ACI), as a promising method of immunotherapy, harnesses the cells that largely expanded *in vitr*o and have intrinsic anti-tumor activity (such as lymphocytes) to eradicate malignant cells [4]. Allogeneic or autologous lymphocytes (autolymphocytes) used by ACI range from tightly defined specificity, e.g., tumor antigen-specific cytotoxic T lymphocytes (CTL) and genetically engineered T cells to broad phenotype and activity, e.g., lymphokine-activated killer (LAK) cells, tumor-infiltrating lymphocytes (TIL) and cytokine-induced killer (CIK) cells, which are heterogeneous effector cell population characterized by co-expression of CD3 and CD56 molecules [5]–[8]. ACI for metastatic renal cell carcinoma (mRCC) was first described in 1990 [9], and then a number of clinical trials with ACI in mRCC patients were completed and the results were released. However, the value of ACI for mRCC remains controversial, especially in tumor regression and prolonging survival. Law [10] et al. and Figlin [11] et al. reported, respectively, that LAK cell or CD8^+^ TIL infusion plus IL-2 did not improve the objective response rate and prolong the survival than IL-2 alone. But Liu [12] et al. reported that CIK cell transfusion significantly improved the objective response rate and the survival than IL-2 plus IFN-α-2a. The aim of this study was to do a systematic review and meta-analysis of published RCTs to investigate the efficacy of ACI in patients with mRCC.

## Methods

### Search Strategy and Selection Criteria

A search of PubMed database (filters activated: RCTs) was conducted until December 12, 2012 using the following keywords: “cytokine induced killer”[Title] OR “tumor infiltrating lymphocytes”[Title] OR “lymphokine activated killer”[Title] OR autolymphocyte[Title] OR “activated T cells”[Title] OR “activated killer cells”[Title] OR “gamma delta T cells”[Title] OR “γδ T cells”[Title] OR “NKT cells”[Title] OR “natural killer”[Title] OR “NK cells”[Title]. Criteria for including studies were treatment of only patients with mRCC, RCTs, ACI *versus* no ACI, and publication in a regular scientific article (exclusion of abstracts).

### Data Extraction and Quality Assessment

Two reviewers (XYT and TL) independently screened the articles identified in the literature search. Disagreements were resolved by a third reviewer (BZ). The following information was collected from each selected article: publication year, number of patients, sex, objective response rate, regimen of ACI, and the number of patients assessable for 1-, 3- and 5-year survival.

The modified 10-point Jadad scale was used to assess the quality of the trials based on the following items, including allocation sequence generation, randomization concealment, methods of blinding, and descriptions of withdrawals and dropouts.

### Statistical Analysis

Statistical meta-analysis was carried out using RevMen 5.2.1 software. Treatment effects are reflected by risk ratios (RR) extracted from objective response, 1-, 3- and 5-year survival. To calculate the pooled RR, the number of responses or survival in each arm was extracted from each study and combined using a method reported by Mantel and Haenszel. A pooled RR>1 indicated higher response or survival rate in ACI arm. To evaluate whether the results of the studies were homogenous, we used the Cochran’s Q test (considering homogeneity for I^2^<50% or P>0.1). The combined RR and 95% CI were calculated with a fixed effect model with no statistically significant heterogeneity existed. All the reported P values were two-sided. P values at <0.05 were regarded as statistically significant.

## Results

**Figure 1:**
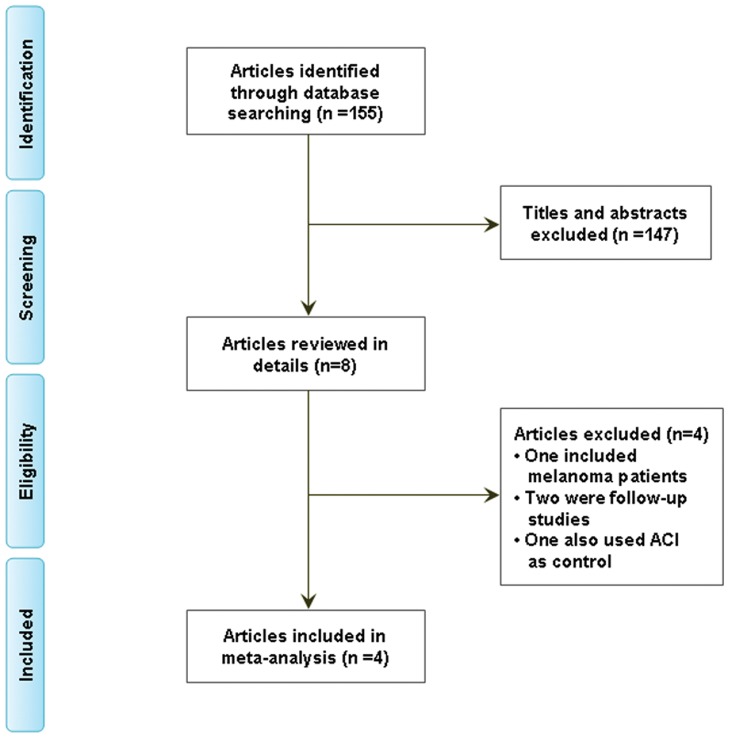
Flow diagram showing record identification, record screening,full text article eligibility and study inclusion process.

4 RCTs, including 469 patients met all selection criteria and were identified (Fig. 1). Of these 4 trials, 3 were conducted in the United States and the remaining one was conducted in China. One trial compared autolymphocyte plus cimetidine with cimetidine alone in mRCC patients; two compared, respectively LAK or CD8^+^ TIL cell plus IL-2 with IL-2 alone in mRCC patients; the other one compared CIK cell alone with IL-2 plus IFN-α-2a in mRCC patients. The characteristics of each trial are shown in Table 1.

**Table 1:** Characteristics of included studies.

Reference	Methods	Patients			Interventions	Outcomes	Phenotypic characteristics of infusion cells
		Eligibility	Control arm	ACI arm			
Osband ME, 1990	RCT; stratifiedby ECOG PS	mRCC with measurable disease; PS (ECOG) 0∼2; suitable haematological indices (packed cell volume>25%, platelet count<500000/µl, and WBC>3000/µl); and no therapy for at least 6 weeks	N = 45; medianage 63 years; PS (0)58%; male 69%	N = 45; median age 64 years;PS (0) 60%;male 69%	(1) 600 mg oral cimetidine (four times daily) with, or (2) without, autolymphocyte therapy *i.v.* approximately 10^9^ cells/month for 6 months	Adverse events; Tumor response; Survival	CD3^+^ (49.6%); CD4^+^ (45.6%); CD8^+^ (18.4%)
Law TM, 1995	Multicenter RCT; stratified by center	mRCC with measurable disease; age 18∼70 years; anticipated survival>16 weeks; karnofsky PS≥80%; SCr≤2.0 mg/dl; SGOT≤150 IU or 4×ULN; SB≤1.6 mg/dl; PTT≤1.5 times control; PT≤1.5 times control; hemoglobin≥10.0 g/dl; AGC≥1500/mm^3^; platelet count≥100000/mm^3^; serum calcium≤12.0 mg/dl; the signing of ICF; absence of brain metastasis or underlying seizure disorder; no history or evidence of significant cardiac dysfunction; no evidence of clinically significant pulmonary dysfunction, pleural effusions, or ascities; no prior treatment with IL-2; and no BRM, radiotherapy, or chemotherapy within previous 4 weeks of entry onto study	N = 36; medianage 53 years;median PS 80;male 86%	N = 35; median age 53 years; median PS 90; male 69%	(1) IL-2 3.0×10^6^ U/m^2^/day (9 mU) *i.v.* on days 1∼5, 13∼17, 21∼24,and 28∼31 with, or (2) without, LAK celltherapy *i.v.* average26.8×10^9^ cells/day ondays 13∼15	Adverse events; Tumor response; Survival	Unknown
Figlin RA, 1999	Multicenter double- blindRCT	RCC with resectable primary tumor; measurable metastatic disease; PS (ECOG) 0∼1; age ≥18 years; willingness to undergo surgey, agreement to use contraception; the signing of ICF; no prior IL-2 therapy, immunotherapy, immunosuppressive therapy, radiotherapy, or chemotherapy within 4 weeks of screening; no significant renal dysfunction (ie, SCr≥2.0 mg/dl), no significant hepatic dysfunction (ie, SB>1.6 mg/dl, ALT>4×normal, and PTT>1.5 control); adequate blood counts (ie, hemoglobin count≥8 g/dl, granulocyte count≥1,500 cells/mm^3^, platelet count≥100,000/mm^3^); no significant cardiovascular disease (ie, heart failure, ischemia, edema, arrhythmia, myocardial infarction, or hypertension); no CNS disease; no pleural effusions or ascites; no active infection; no active peptic ulcer disease; no antibodies to HIV, hepatitis B surface antigen, or hepatitis C; no only bone or abdominal metastases; no prior history of malignancy within the last 5 years other than basal cell carcinoma or cervical carcinoma-in-situ; serum calcium level ≤12 mg/dl or no symptomatic hypercalcemia; no use of corticosteroids or calcium channel and beta adrenergic blockers; women who were not pregnant and/or nursing; no solitary kidney; insignificant intercurrent illnesses and no New York Heart Association class III or IV	N = 79; medianage 55 years;PS (0) 44.3%;male 67.1%	N = 81; median age 56 years; PS (0) 46.9%; male 86.4%	(1) IL-2 5×10^6^ IU/m^2^/day *i.v.* for 4 days for 4 weeks with, or (2) without a single *i.v.* infusion of (5∼3000)×10^7^ CD8^+^ TIL cells	Adverse events; Tumor response; Survival	CD3^+^ (91.8±17.4%); CD4^+^ (9.0±17.8%); CD8^+^ (84.8±23.3%); CD56^+^ (30.1±22.0%); CD3^+^CD8^+^ (76.6±32.8%); CD8^+^CD56^+^ (22.3±19.6%)
Liu L, 2012	RCT; stratified by a block method	progressive and irresectable metastatic clear cell RCC; anticipated survival>3 months; karnofsky PS>40%; age 18∼80 years; SB and SCr less than 1.25×ULN, and free of congestive heart failure, severe coronary artery disease, cardiac arrhythmias, HIV infection, chronic active hepatitis and concomitant corticosteroid therapy; no chemotherapy or immunotherapy during the previous 4 weeks; and women who were not pregnant and lactating	N = 74; medianage 60 years;karnofsky PS(≥80) 43%;male 82%	N = 74; median age 59 years; karnofsky PS (≥80) 38%;male 81%	(1) IL-2 (10×10^6^ IU/m^2^/day *s.c.* on day 1, 3 and5, weeks 1∼4) plusIFN-α-2a (3×10^6^ IU/m^2^/day*s.c.* on day 1, 3 and 5,weeks 1∼4) versus (2)CIK cell therapy *i.v.*average 9.7×10^9^ cells/cyclefor at leastone cycle	Adverse events; Tumor response; Survival	CD3^+^ (81.06±9.22%); CD3^+^CD4^+^ (42.70±6.18%); CD3^+^CD8^+^ (36.41±5.19%); CD3^+^CD56^+^ (18.21±4.73%); CD25^+^ (33.13±6.87%)

AGC: absolute granulocyte count, BRM: biologic response modifier, ECOG: Eastern Cooperative Oncology Group, ICF: informed consent forms, PT: prothrombin time, PTT: partial thromboplastin time, PS: performance status, SB: serum bilirubin, SCr: serum creatinine, SGOT: serum glutamic oxaloacetic transaminase, ULN: upper limit of normal, WBC: while blood cell.

The Jadad score was 3 for one trial and 4 for the other three.

### Toxicity

The distributions of side effects in ACI and control groups were reported in all 4 trials. In general, ACI was generally well tolerated and most of ACI-related adverse reactions were grade 1 or 2 and reversible without additional treatment. However, one trial demonstrated that IL-2 plus LAK cell resulted in more pulmonary toxicity (P = 0.008) and hypotensive episodes (P = 0.051) compared with IL-2 alone [10]. Another trial indicated that the incidences of three toxicities (embolus, apnea and dyspnea) caused by IL-2 plus TIL cell were at least twice than that in IL-2 alone group [11].

### Objective Response

**Figure 2:**
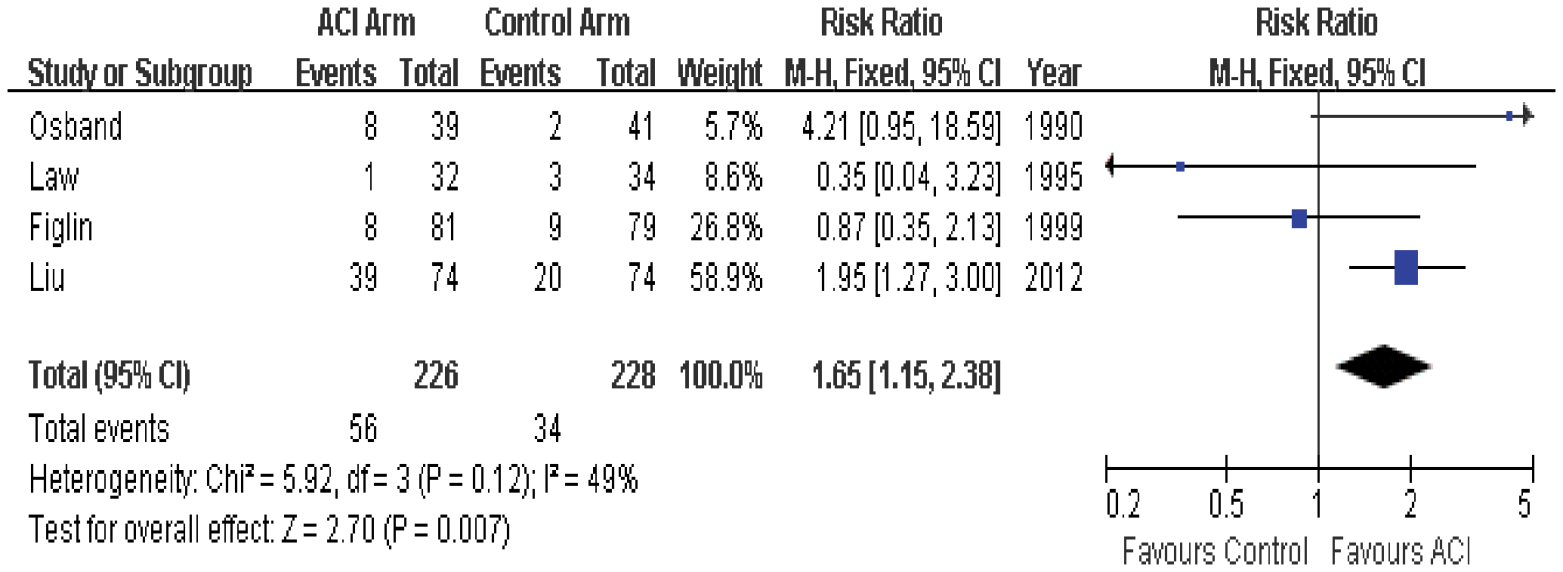
Comparison of objective response between ACI and control (no ACI) groups.

Tumor response data was available in all 4 trials. 2 trials reported that objective response rate in ACI group was significantly better than that in the control group, while the other 2 trials did not find differences between the two groups. The estimated pooled RR for all 4 trials shows a highly significant improvement of objective response for patients with ACI or not (RR = 1.65; 95% CI, 1.15 to 2.38; P = 0.007; Fig. 2). The Cochran’s Q test had a P value of 0.12 and the corresponding quantity I^2^ was 49%, indicating that the heterogeneity among individual studies was acceptable.

### 1-year Survival

**Figure 3:**
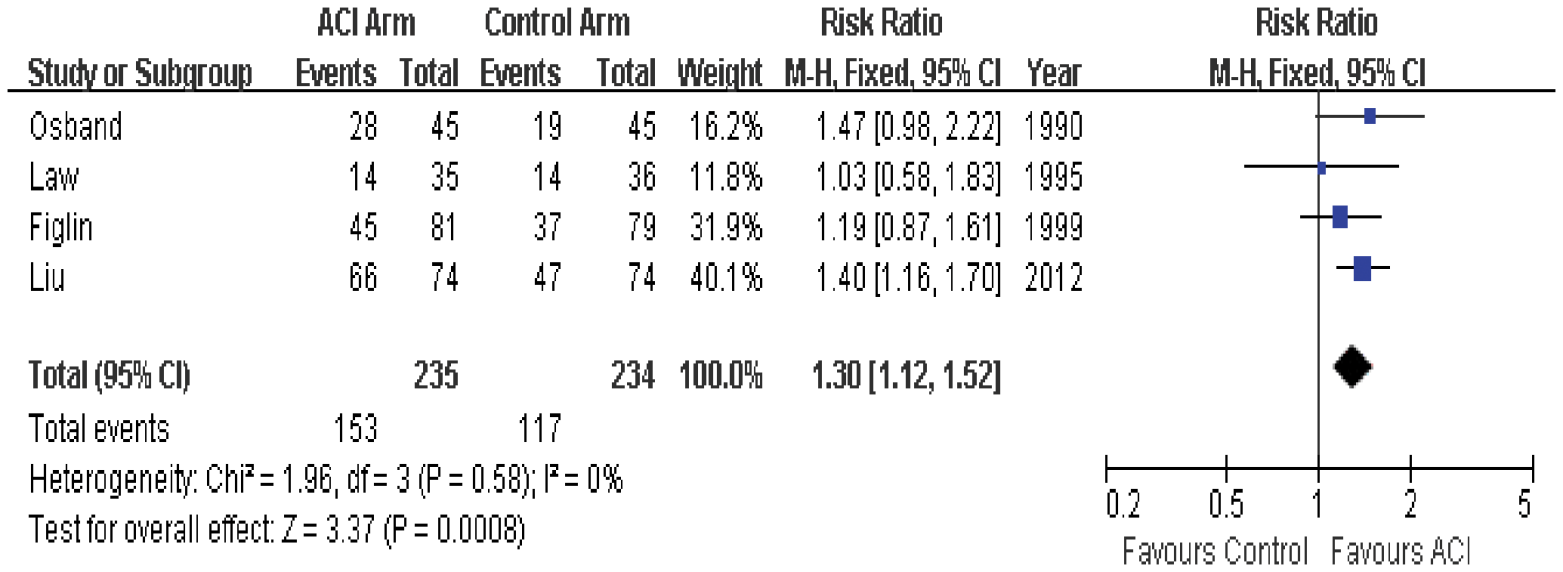
Comparison of 1-year survival between ACI and control (no ACI) groups.

1-year survival data was available in all 4 trials. Two of trials shown that 1-year survival rate in ACI group was significantly better than that in the control group, while the other 2 trials did not found difference between the two groups. The estimated pooled RR for all 4 trials shows a highly significant improvement of 1-year survival for patients with ACI or not (RR = 1.30; 95% CI, 1.12 to 1.52; P = 0.0008; Fig. 3). The Cochran’s Q test had a P value of 0.58 and the corresponding quantity I^2^ was 0%, indicating that there was no evidence of heterogeneity among individual studies.

### 3-year Survival

**Figure 4:**
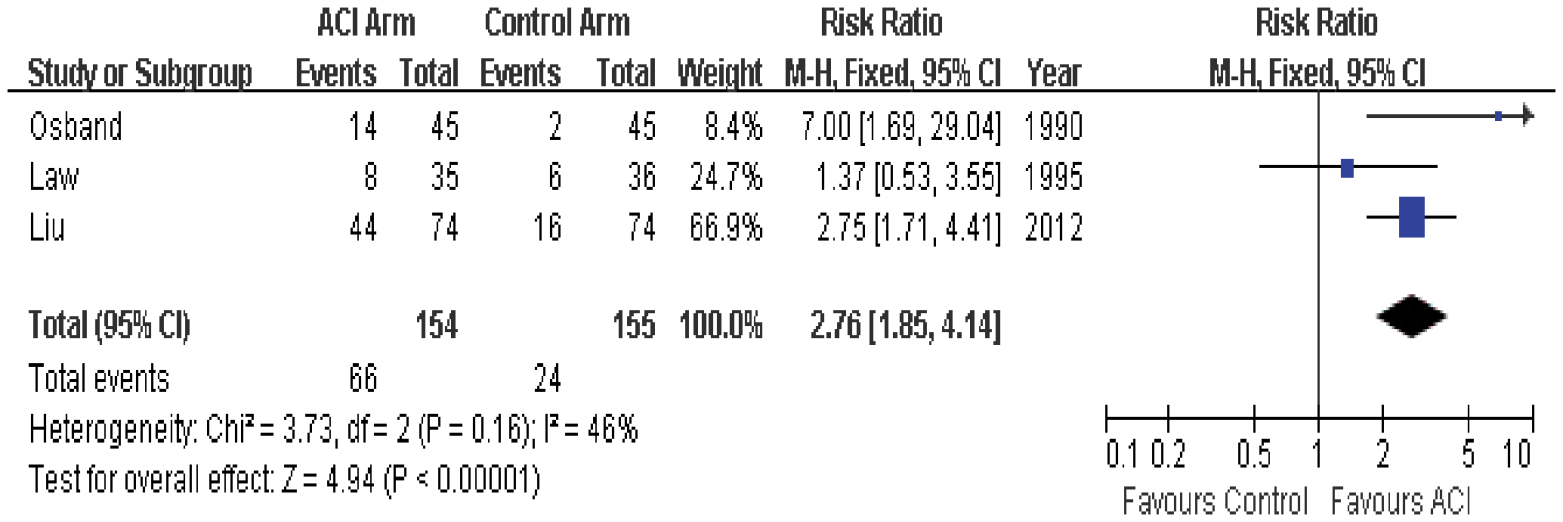
Comparison of 3-year survival between ACI and control (no ACI) groups.

3-year survival data was available in only 3 trials. Two of trials indicated that 3-year survival rate in ACI group was significantly better than that in the control group, while the other one trial had similar 3-year survival rate between the two groups. The estimated pooled RR for 3 trials demonstrates that ACI can significantly improve the 3-year survival in patients with mRCC (RR = 2.76; 95% CI, 1.85 to 4.14; P<0.00001; Fig. 4). The Cochrane’s Q test had a P value of 0.16 and the corresponding quantity I^2^ was 46%, indicating that the heterogeneity among individual studies was acceptable.

### 5-year Survival

**Figure 5:**
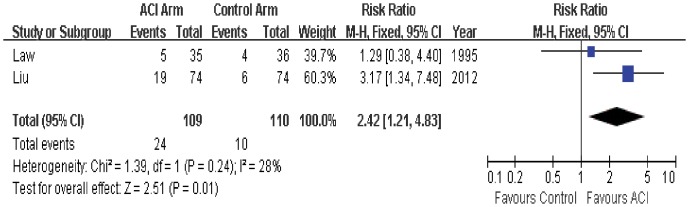
Comparison of 5-year survival between ACI and control (no ACI) groups.

5-year survival data was available in only 2 trials. One trial showed that 5-year survival rate in ACI group was significantly better than that in the control group, while another trial did not found difference between the two groups. The estimated pooled RR for 2 trials indicates that ACI can significantly improve the 5-year survival in the patients with mRCC (RR = 2.42; 95% CI, 1.21 to 4.83; P = 0.01; Fig. 5). The Cochrane’s Q test had a P value of 0.24 and the corresponding quantity I^2^ was 28%, showing that the heterogeneity among individual studies was acceptable.

## Discussion

RCC is a relatively rare disease accounting for 2∼3% of all malignancies in adults. There are approximately one third of RCC patients has metastatic disease at diagnosis and 30∼50% of initially localized RCC eventually metastasize [13]. A number of desirable ACI has been developed to improve the survival of mRCC patients because the median survival of mRCC patients is only 10.2 months [13], 5-year survival rate is less than 15% [14] and it has been shown that RCC is an immunogenic tumor with response to systemic cytokine therapy, occasional regressions and frequent leukocytic infiltration [13]. However, the value of ACI for mRCC is still unclear. Therefore, this systematic review sought to identify all types of ACI for mRCC and do a meta-analysis. As shown above, our analysis demonstrates that ACI may be a safe, effective treatment that can improve objective response, 1-, 3- and 5-year survival for mRCC.

Two main reasons for the differences between our conclusions and the ones of some original studies are listed as follows. For one thing, one trial focused on clear cell RCC, but the remaining three did not identify subtypes of RCC. It has been shown that RCC has three common histological subtypes, including clear cell (70∼80%), papillary (10∼15%) and chromophobe (3∼5%) [15], and clear cell RCC appears to be the only histological subtype, which is responsive to immunotherapy [16]. For another, autolymphocytes, LAK, CD8^+^TIL or CIK cells may have different effect on mRCC. Although mechanisms of anti-tumor action of these cell products are all based on cytotoxic effects of lymphocytes, especially T lymphocytes and NK cells, cell characteristics (e.g. cell dosage and cell phenotype) vary greatly from one infusion product to another and it may result in variable anti-tumor effect [17]. Take T lymphocyte phenotype for example, one trial did not report cell phenotype of transfusion cells and percentage of T lymphocytes of cell products in other three trials are 49.6%, 91.8±17.4% and 81.06±9.22%, respectively (Table 1). Therefore, our analysis has different conclusions in objective response and survival comparing with the ones from some original trials.

There are three major limitations in our study. To begin with, our analysis was based on published data from 4 RCTs with different ACI protocols. As mentioned above, the clinical effect of ACI on cancer treatment relies on cytotoxicity mediated by T lymphocytes or NK cells and is determined in part by the infusion dosage of these cytotoxic cells. But data on cell dosage and phenotype of infusion products were variable (Table 1). In addition, ACI groups of two studies also used IL-2 and it may improve the objective response because IL-2 has been shown to induce objective response in patients with RCC [18]. Moreover, the Jadad scores of included trials were 3 and 4. Some studies did not clearly report randomization, allocation concealment, blinding or withdrawals and dropouts, and it may lead to distribution and implementation bias in current meta-analysis. Finally, there were race differences between patients in this meta-analysis. Three RCTs were conducted in the United States, while the remaining one was in China. Therefore, the reliability of this systematic review and meta-analysis might be influenced by these factors and the results have to be interpreted with caution.

ACI has been introduced in the clinic for *several decades* and has proven to be feasible, less-toxic and effective in some patients, especially leukemia [19], melanoma [20], hepatocellular carcinoma [21] and RCC. However, ACI has not yet been approved as a standard treatment for solid tumors due to some obstacles to achieving efficacy. First, ACI is highly personal and doesn’t fit into the paradigm of drug development. As we all know, a drug is created for every patient and can be used off-the-shelf, but ACI uses a patient’s own cells to treat them and must be used in a personalized way [22]. On the other hand, the personalized ACI means high expense, for example, the TIL cell production costs range from $20,000 to $25,000 per patient, and leads to less enthusiasm for investment from biotech companies [22]–[23]. Thus, collaborative efforts between biotech companies, blood banks and academic institutions will enhance the development of ACI. Second, objective response induced by ACI is traditionally assessed by WHO or RECIST criteria, but these criteria may not be suitable for ACI since they were developed based on chemotherapy. ACI and chemotherapy have completely different mechanisms for fighting cancer. The former exerts its effect on immune system and is characterized by a new kinetics based on cellular immune response followed by changes in tumor burden or survival time, while the latter acts directly on tumor cells and results in tumor shrinkage in a few weeks of initial administration [24]. A novel immune-related response criteria (irRC) was already recommended to assess immunotherapy clinical activity by Cancer Research Institute Cancer Vaccine Consortium in 2009 [25]. Third, identification of TAAs, especially patient-specific tumor antigens is inadequate. Most current effector cell populations used for ACI are not tumor antigen specific (e.g. LAK or CIK cells) due to lack of TAAs. This problem might be addressed by routine DNA sequencing techniques in the future [26]. Fourth, methods for improving ACI are required to integrate into clinical protocols, for example, prior lymphodepletion. According to Rosenberg SA’s experiences in advanced melanoma, lymphodepletion before TIL cell transfer is essential because it can get rid of regulatory elements (e.g. regulatory T cells or myeloid-derived suppressor cells) in tumor microenvironment and eliminate endogenous lymphocytes that consume homeostatic cytokines like IL-7 and IL-15, which are responsible for sustaining transferred TIL cell survival [26]. Fifth, physician's understanding of cell quality of ACI-based products is inadequate. Some studies did not report quality controls, which are composed of microbiological controls and purity, morphology, phenotype, number and viability of infusion cells in detail [10]. Therefore, we are unable to compare directly the results of individual studies and unable to optimize the protocols with ACI. In summary, the above obstacles to successful ACI should be overcome. Only after resolving these problems, can ACI finally be made available to patients with solid tumor as a standard option.

## Supporting Information

Checklist S1 PRISMA checklist.
(DOC)
